# The J-Domain of Heat Shock Protein 40 Can Enhance the Transduction Efficiency of Arginine-Rich Cell-Penetrating Peptides

**DOI:** 10.1155/2015/698067

**Published:** 2015-05-17

**Authors:** Tzu-Yin Lin, Yu-Hsiu Su, Kun-Hsiung Lee, Chin-Kai Chuang

**Affiliations:** ^1^Division of Animal Technology, Animal Technology Laboratories, Agricultural Technology Research Institute, No. 1, Lane 51, Dahu Road, Xiangshan District, Hsinchu 30093, Taiwan; ^2^Division of Biotechnology, Animal Technology Institute Taiwan, No. 52, Kedong 2nd Road, Chunan, Miaoli 35059, Taiwan

## Abstract

Sense and antisense oligonucleotide pairs encoding cell-penetrating peptides PTD (Tat_47–57_), DPV3A, E162, pVEC, R11, and TP13 were used to construct two sets of pET22b-CPP-DsRed and pET22b-CPP-J-DsRed vectors for CPP-DsRed and CPP-J-DsRed recombinant proteins expression. PTD-DsRed, DPV3A-DsRed, PTD-J-DsRed, and DPV3A-J-DsRed recombinant proteins were expressed in a soluble form. PTD-J-DsRed and DPV3A-J-DsRed recombinant proteins were able to escape from *E. coli* host cells into the culture medium. The membrane-penetrating activity of PTD-J-DsRed and DPV3A-J-DsRed recombinant proteins to mammalian cells was more effective than that of PTD-DsRed and DPV3A-DsRed. The route of the cellular membrane translocation of these recombinant proteins is suggested via macropinocytosis followed by an endosomal escape pathway.

## 1. Introduction

### 1.1. Cell-Penetrating Peptides (CPPs)

Since the observation that HIV-1 Tat protein could shuttle between cells and the discovery that purified Tat protein could enter cells and translocate into nuclei [1], the cell-penetrating activity of Tat has been narrowed down gradually from amino acids 36–72 [2], to either amino acids 48–60 [3] or amino acids 47–57 [4]. The protein transduction domain (PTD, specifically indicating the peptide: Tat amino acids 47–57, hereafter) of Tat protein was able to deliver macromolecule, such as 120 kDa *β*-galactosidase, fused to it* in vivo* [5, 6]. Meanwhile, a 16-amino acid peptide derived from the third helix of the homeodomain of Antennapedia, termed as penetratin, was found to translocate through cell membrane as well [7]. Up to now, a lot of cell-penetrating peptides (CPPs) have been reported (for review, see [8]) and the information has been collected and compiled in a website [9]. CPPs, either protein-derived or chemically synthesized, can be categorized into primary amphipathic, secondary amphipathic, and nonamphipathic [10]. The primary amphipathic CPPs such as transportan [11] and TP10 [12] are usually longer than 20 amino acids with periodically hydrophobic and hydrophilic residues along the primary sequence. In comparison to the primary amphipathic CPPs, the secondary amphipathic CPPs such as penetratin, pVEC [13], and E162 [14] contain less amino acid residues and perform amphipathic structure upon interacting with phospholipid membrane. The third class CPPs, such as R8 [15], DPV3 [16], and PTD [4], are relatively short and contain very high content of arginine.

### 1.2. Membrane-Penetration Mechanisms of Arginine-Rich CPPs

A metabolic energy-independent, direct plasma membrane translocation mechanism could be detected for the highly positively charged R8 peptide at 4°C at which the receptor-mediated internalization was completely inhibited. However, only a small part of R8 penetration occurred by way of this pathway at physiological temperature [17]. Pyrenebutyrate can neutralize the positive charge of R8 [15], R9, and Tat_48–60_ [18] and provide a hydrophobic aromatic group to accelerate the direct penetration through the cellular membrane. At 37°C, the translocation of arginine-rich CPPs into cytoplasm is mainly via an endocytic uptake-endosomal escape pathway. The translocation of arginine-rich CPPs is not dependent on both clathrin and caveolin-coated pit-mediated endocytosis but is inhibited by 5-(N-ethyl-N-isopropyl)amiloride (EIPA), an inhibitor of macropinocytosis [19], and cytochalasin D, which prevents actin polymerization [20]. Membrane-associated proteoglycan including heparin sulfate (HSPG) is reported to play a crucial role in the endocytic uptake of arginine-rich CPPs [21]. It could be concluded that the positively charged arginine-rich CPPs associated with negatively charged proteoglycan on the surface of plasma membrane were engulfed by macropinocytosis followed by endosomal escape into the cytoplasm. The endosomal escape step is rate limiting for the CPPs to arrive at the cytosol; however, the mechanism is not well explored.

### 1.3. PTD-J-Domain

Recently, a vector pET22b-PTD_1_J_1_ that could be used to highly express recombinant protein fused to PTD-J-domain on its N-terminus was reported. We took advantages of the specific association ability of the J-domain of Hsp40 with the nucleotide binding domain of Hsp70 and the cell membrane-penetrating activity of the protein transduction domain of HIV-1 Tat protein. Higher level and more soluble chicken IGF-I recombinant protein was expressed by the pET22b-PTD_1_J_1_ vector in comparison to the pET32b vector. An HpNC peptide containing two fragments of human heptoprotein was expressed by the pET22b-PTD_1_J_1_ vector. The PTD-J-HpNC recombinant polypeptide product could effectively elicit rat antisera specific to subtypes Hp1 and Hp2 heptoproteins in human serum samples, but the counterpart TrxA-HpNC could not [22]. Moreover, overexpression of PTD-J-FMDVepi, where FMDVepi is an assembled T_H_ and B-epitopes of foot-and-mouth disease virus VP1 capsid protein, is dependent on the combination of PTD and J-domain rather than PTD or J-domain individually. This result suggests that the fused PTD-J polypeptide may possess a special structure that can elicit the immunogenicity of FMDVepi peptide fused with it [23].

In this study, two sets of pET22b-CPP and pET22b-CPP-J expression vectors were constructed. The CPP-DsRed and CPP-J-DsRed recombinant proteins expressed by them were characterized. The cellular membrane-penetrating capabilities of the chosen CPPs were elevated by the J-domain fused to them.

## 2. Materials and Methods

### 2.1. Construction of pET22b-CPP-DsRed and pET22b-CPP-J-DsRed Vectors

The coding region of the red fluorescence protein in pDsRed monomer N1 plasmid (Cat. no. 632465, Clontech) was amplified by PCR with forward primer (G AAT TCT CAT ATG ATG GAC AAC ACC GAG GAC GTC ATC) and reverse primer (CTC GAG ACC ACC CTG GGA GCC GGA GTG GCG GGC CT). The amplified DNA fragment was ligated with pGEM TEasy TA-cloning vector (A1360, Promega) to get pGEM TE-DsRed. Then, the cloned DsRed DNA fragment was removed from the cloning vector by EcoRI and XhoI restriction enzyme digestion and subcloned to the pET22b-PTD_1_J_1_ expression vector [22] which had been treated by the same pair of restriction enzymes to obtain pET22b-PTD-J-DsRed plasmid. The sense and antisense oligonucleotide pairs of PTD, DPV3A (the first amino acid residue R of DPV3 was replaced by A), E162, pVEC, R11, and TP13 (Table 1) were annealed in TEN (10 mM Tris-HCl, pH 8.0/1 mM EDTA, pH 8.0/0.3 M NaCl) at 60°C for 30 min followed by slowly cooling down to room temperature for about 60 min. These annealed primer pairs were inserted between NdeI and EcoRI sites of pET22b to obtain pET22b-PTD, pET22b-DPV3A, pET22b-E162, pET22b-pVEC3, pET22b-R11, and pET22b-TP13. The DsRed DNA fragment described above was inserted between the EcoRI and XhoI sites of pET22b-PTD, pET22b-DPV3A, pET22b-E162, pET22b-pVEC3, pET22b-R11, and pET22b-TP13 to get pET22b-PTD-DsRed, pET22b-DPV3A-DsRed, pET22b-E162-DsRed, pET22b-pVEC3-DsRed, pET22b-R11-DsRed, and pET22b-TP13-DsRed, respectively. The J-DsRed fragment removed from pET22b-PTD-J-DsRed by BamHI and XhoI codigestion was inserted between BamHI and XhoI sites of pET22b-DPV3A, pET22b-E162, pET22b-pVEC3, pET22b-R11, and pET22b-TP13 to get pET22b-DPV3A-J-DsRed, pET22b-E162-J-DsRed, pET22b-pVEC3-J-DsRed, pET22b-R11-J-DsRed, and pET22b-TP13-J-DsRed, respectively.

### 2.2. Construction of pET22b-DsRed and pET22b-J-DsRed Vectors

The 0.7 kb NdeI-XhoI fragment of pGEM TE-DsRed clone was inserted into the same restriction enzyme sites of pET22b to obtain pET22b-DsRed. The primer pair, CAT ATG GGT AAA GAT TAC TAC CAG ACT CAC GGT and GA ATT CGA ACC ACG TGG AAC TAA ATT CGC ACC ACC AGA, was used to amplify DNA fragment encoding the J-domain and a thrombin cutting site using pET22b-PTD_1_J_1_ as template. This DNA fragment was utilized to replace the PTD-J fragment which is franked by NdeI and EcoRI sites of the pET22b-PTD_1_J_1_-DsRed vector to prepare pET22b-J-DsRed.

### 2.3. Expression and Purification of CPP-DsRed, CPP-J-DsRed, DsRed, and J-DsRed Recombinant Proteins

The* E. coli* Rosetta gamiB(DE3)pLysS host cells transformed by pET22b-CPP-DsRed, pET22b-CPP-J-DsRed, pET22b-DsRed, or pET22b-J-DsRed were grown in 2x YT supplemented with 0.4% glucose, 30 *μ*g/mL chloramphenicol, and 50 *μ*g/mL ampicillin at 37°C. IPTG was adjusted to 1 mM when OD_600_ was 0.6 and cells were cultured for another 4 h. To analyze recombinant proteins released into the medium, cells were centrifuged at 12,000 g for 30 min. The supernatant was concentrated 10-fold using Centricon (Y3, Millipore), and then 30 *μ*L of sample was loaded in each lane of a 12% SDS polyacrylamide gel. To analyze recombinant proteins within cells, the cells were collected by centrifugation at 10,000 g for 10 min. After ultrasonication, protein contents of soluble fraction and insoluble fraction corresponding to 0.2 OD_600_ unit of cells were run on a 12% SDS polyacrylamide gel. The gel was stained with Coomassie Blue R250. Soluble forms of CPP-DsRed and CPP-J-DsRed recombinant proteins were purified by Ni-Sepharose 6 Fast Flow affinity column (17-5318-02, GE) in accordance with the manufacture's instruction.

### 2.4. Protein Transduction

Huh-7 cells were seeded at 1.5 × 10^5^ cells per well in a 24-well plate and cultured in DMEM supplemented with 10% FBS the day before protein transduction experiment. The cells were washed with serum-free medium twice and incubated with various concentrations of recombinant proteins in serum-free medium for various times as indicated. The unpenetrated recombinant proteins were washed off with PBS twice. Then, the penetrated proteins were released from cells using 200 *μ*L of PBS supplemented with 1% Triton X-100. After centrifugation at 10,000 g for 5 min to remove the nonsoluble materials, 100 *μ*L supernatant was transferred to a well of a 96-well plate to measure the amount of recombinant DsRed proteins in the soluble fraction using a fluorometer. The stimulating wave length and emission wave length were set at 557 nm and 585 nm, respectively. Experiments were repeated for four times. The recombinant DsRed proteins penetrated into cells were also detected with a fluorescence microscope. The calibration curves between the fluorescence values of 100 *μ*L sample per well and the concentrations of recombinant proteins in total nontransduced cell lysate were measured as described above. To test the effects of endocytosis inhibitors on the cell-penetration activity of PTD-J-DsRed, cells were pretreated with filipin (5 *μ*g/mL; Sigma, F9765), EIPA (100 *μ*M; Sigma, A3085), or cytochalasin D (10 *μ*M; Sigma, C8273) for 1 hour before the treatment of the PTD-J-DsRed recombinant protein (40 *μ*g/mL) for two hours.

## 3. Results

### 3.1. Construction of pET22b-CPP-DsRed and pET22b-CPP-J-DsRed Expression Vectors

**Figure 1:**
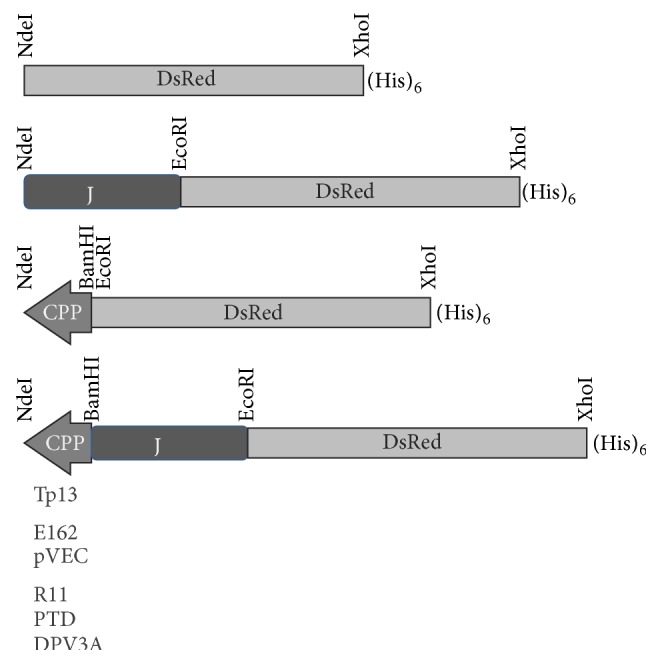
Representative structures of the CPP-DsRed and CPP-J-DsRed. Oligonucleotide pairs encoding DPV3A, E162, pVEC, R11, and TP13 listed in Table 1 were inserted between NdeI and EcoRI sites of pET22b to create pET22b-DPV3A, pET22b-E162, pET22b-pVEC, pET22b-R11, pET22b-PTD, and pET22b-TP13, respectively. The DsRed cDNA fragment cut from pET22b-PTD-J-DsRed by the EcoRI site at 5′-end and the XhoI site at 3′-end was inserted into the above vectors to prepare pET22b-DPV3A-DsRed, pET22b-E162-DsRed, pET22b-pVEC-DsRed, pET22b-R11-DsRed, pET22b-PTD-DsRed, and pET22b-TP13-DsRed. The J-DsRed cDNA fragment cut from pET22b-PTD-J-DsRed by the BamHI site at 5′-end and the XhoI site at 3′-end was inserted into the above vectors to prepare pET22b-DPV3A-J-DsRed, pET22b-E162-J-DsRed, pET22b-pVEC-J-DsRed, pET22b-R11-J-DsRed, and pET22b-TP13-J-DsRed.

Published CPPs with better transduction activities were focused and TP13 (primary amphipathic CPP) [24], E162, and pVEC (secondary amphipathic CPPs) [14], as well as R11 [25], PTD [22], and DPV3 [16] (arginine-rich CPPs), were picked up in this study. The pET22b-CPP-DsRed and pET22b-CPP-J-DsRed expression vectors were constructed as described in Section 2 and the representative structures of CPP-DsRed and CPP-J-DsRed accompanied with the DsRed and J-DsRed controls are shown in Figure 1.

### 3.2. Expression of CPP-DsRed Recombinant Proteins

**Figure 2:**
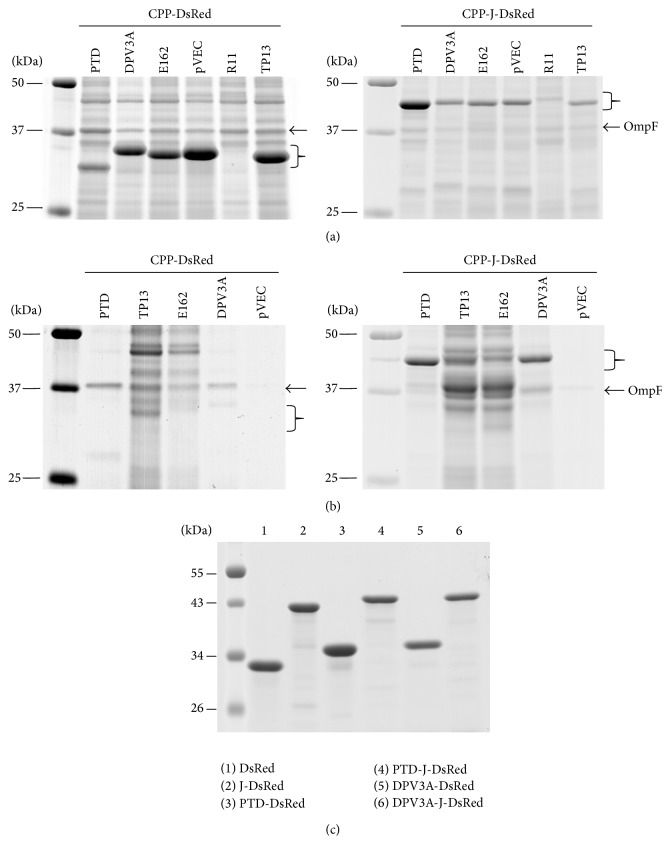
Expression of CPP-DsRed recombinant proteins. (a) Total lysates of* E. coli* expressing PTD-DsRed, DPV3A-DsRed, pET22b-E162-DsRed, pVEC-DsRed, R11-DsRed, and TP13-DsRed recombinant proteins (left panel) as well as those of PTD-J-DsRed, DPV3-J-DsRed, E162-J-DsRed, pVEC-J-DsRed, R11-J-DsRed, and TP13-J-DsRed recombinant proteins (right panel) were analyzed by SDS-PAGE. The recombinant proteins are indicated by braces. (b) The protein contents of medium fractions from* E. coli *expressing PTD-DsRed, DPV3A-DsRed, E162-DsRed, pVEC-DsRed, and TP13-DsRed recombinant proteins (left panel) as well as those of PTD-J-DsRed, DPV3-J-DsRed, E162-J-DsRed, pVEC-J-DsRed, R11-J-DsRed, and TP13-J-DsRed (right panel) were analyzed by SDS-PAGE. A 38 kDa common secreted protein OmpF from host cells is indicated by an arrow as internal standard and the recombinant proteins are indicated by braces. (c) The soluble DsRed, J-DsRed, PTD-DsRed, PTD-J-DsRed, DPV3A-DsRed, and DPV3A-J-DsRed recombinant proteins were purified by Ni-Sepharose 6 Fast Flow affinity column chromatography. Each lane was loaded with 20 *μ*g of purified recombinant proteins.

After transformation with pET22b-CPP-DsRed plasmids,* E. coli *Rosetta gamiB(DE3)pLysS cells were cultured in 2x YT medium supplemented with 0.4% glucose and antibiotics until OD_600_ reached 0.6. Then, IPTG was adjusted to 1 mM and cells were cultured for another 4 h. The expression level of R11-DsRed recombinant protein in the total lysate was nearly undetectable (left panel of Figure 2(a)); therefore, only the other five recombinant proteins were characterized in the following steps. After homogenization by ultrasonication, the cell lysate can be separated into soluble (supernatant) and insoluble (pellet) fractions by centrifugation. PTD- and DPV3A-DsRed recombinant proteins were found in the soluble fraction; on the other hand, TP13-, E162-, and pVEC-DsRed recombinant proteins were found in the insoluble fraction (data not shown). The growth rates of the TP13- and E162-DsRed cultures were slower. After removal of cells by centrifugation and 0.22 *μ*m membrane filtration, the protein contents in medium after IPTG induction were analyzed by SDS-PAGE. As the patterns shown in the left panel of Figure 2(b), large amounts of cellular proteins of* E. coli* were detected in the TP13- and E162-DsRed lanes; however, a 38 kDa protein band was dominantly detected (indicated by an arrow) in the PTD- and DPV3A-DsRed lanes. These results indicated that TP13- and E162-DsRed recombinant proteins might disturb the cell membrane of* E. coli* host cells and cause cell disruption, even if most of them were present in the insoluble form.

### 3.3. Expression of CPP-J-DsRed Recombinant Proteins

The expression characteristics of PTD-J-, DPV3A-J-, TP13-J-, E162-J-, and pVEC-J-DsRed are similar to those of PTD-, DPV3A-, TP13-, E162-, and pVEC-DsRed, respectively (right panel of Figure 2(a)). The major difference is that significant amounts of PTD-J-DsRed and DPV3-J-DsRed recombinant proteins were presented in the medium fractions (right panel of Figure 2(b)). These two “secreted” forms of recombinant proteins could be purified by Ni-NTA affinity column and contain N-terminal amino acid sequences the same as those of PTD-J-DsRed and DPV3A-J-DsRed indicating that they have intact N- and C-termini. In comparison with the amount of recombinant proteins that remain within cells, about 10% of these two recombinant proteins were in the secreted fractions.

### 3.4. Cell-Penetrating Activities of PTD-, DPV3A-, PTD-J-, and DPV3A-J-DsRed Recombinant Proteins

Recombinant DsRed, J-DsRed, PTD-DsRed, PTD-J-DsRed, DPV3A-DsRed, and DPV3A-J-DsRed proteins (Figure 2(c)) were purified from cell lysates by Ni-NTA-Sepharose affinity column chromatography. After elution by 250 mM imidazole, these proteins were dialyzed against 100 volumes of PBS twice, and aliquots were stored at −80°C.

**Figure 3:**
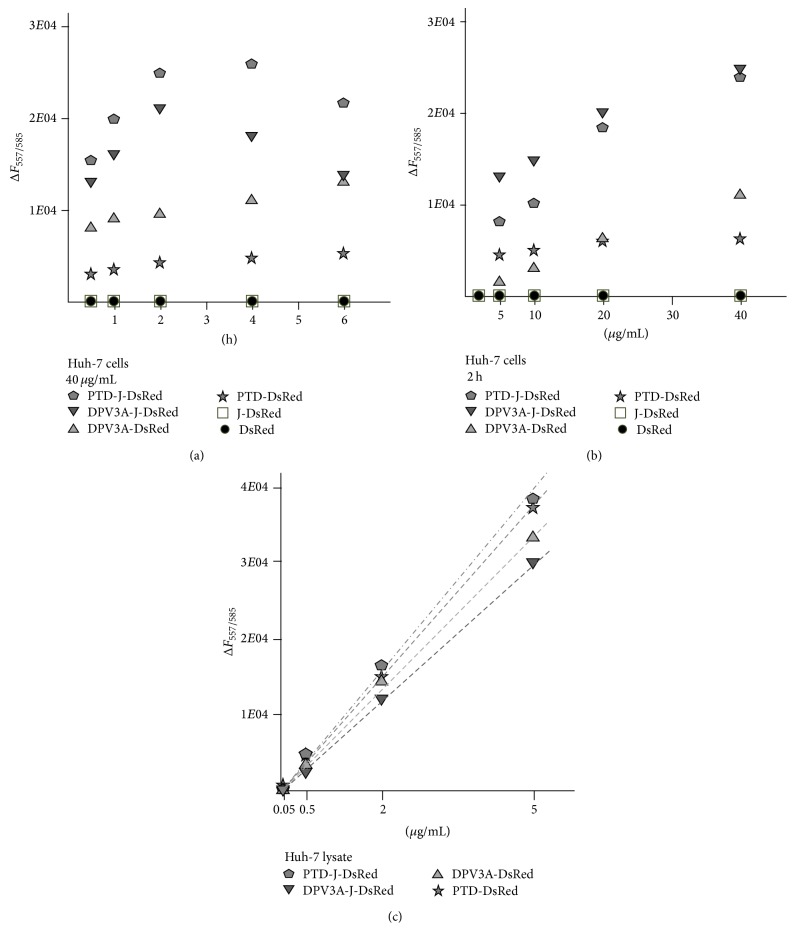
Transduction of PTD-DsRed, DPV3A-DsRed, PTD-J-DsRed, and DPV3A-J-DsRed recombinant proteins. (a) 1.5 × 10^5^ Huh-7 cells in a well of 24-well plate were incubated with 0.2 mL of PTD-DsRed, DPV3A-DsRed, PTD-J-DsRed, or DPV3A-J-DsRed recombinant protein (40 *μ*g/mL) for 0.5, 1, 2, 4, or 6 h. The cells were washed with PBS twice and the recombinant proteins incorporated into cells were released by 0.2 mL of 1% Triton X-100/PBS. After centrifugation to remove cell debris, 0.1 mL of the supernatant was transferred to a well of a 96-well plate and the amounts of recombinant proteins were measured by a fluorometer. The stimulating wave length and emission wave length were set at 557 nm and 585 nm, respectively. (b) 1.5 × 10^5^ Huh-7 cells in a well of 24-well plate were incubated with 0.2 mL of 5, 10, 20, and 40 *μ*g/mL of the PTD-DsRed, DPV3A-DsRed, PTD-J-DsRed, or DPV3A-J-DsRed recombinant protein for 2 hours. The amounts of recombinant proteins were measured as described before. Experiments were done for four times and the average values were shown. All of the standard deviations were less than 1000. (c) The PTD-DsRed, DPV3A-DsRed, PTD-J-DsRed, and DPV3A-J-DsRed recombinant proteins were diluted in Huh-7 cell lysate to 0.05, 0.5, 2, and 5 *μ*g/mL and the fluorescence values were measured as described above.

The cell-penetrating activities of the four recombinant proteins were tested. At first, the time course of recombinant protein transduction to Huh-7 cells was measured at 0.5 1, 2, 4, and 6 hours using 40 *μ*g/mL of each recombinant protein in serum-free Opti-MEM medium. Both of the DsRed and J-DsRed could not penetrate into Huh-7 cells. The amount of the PTD-DsRed and DPV3A-DsRed proteins transduced increased with time up to 6 hours. However, the time for optimal amount of PTD-J-DsRed and DPV3A-J-DsRed transduced into cells was around 2 to 4 hours (Figure 3(a)). Then, Huh-7 cells were treated with 5, 10, 20, and 40 *μ*g/mL of each recombinant protein in the same medium for 2 hours. The amounts of recombinant proteins incorporated increased roughly proportional to the amounts of the recombinant proteins added (Figure 3(b)). To estimate the amounts of recombinant proteins transduced into cells, calibration curves of the fluorescence values of 100 *μ*L sample per well relative to the concentrations of recombinant proteins in total cell lysate were illustrated in Figure 3(c). According to the calibration curves, about 2% of PTD-DeRed and DPV3A-DsRed and 7% of PTD-J-DeRed and DPV3A-J-DsRed can transduce into cells when 40 *μ*g/mL of recombinant proteins was used.

**Figure 4:**
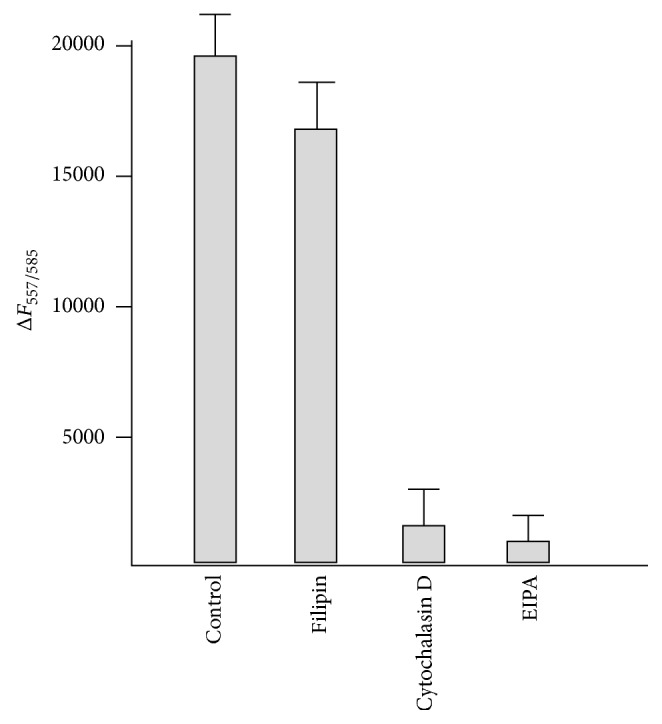
Macropinocytosis is involved in the cell-penetration pathway of PTD-J-DsRed. Huh-7 cells were pretreated with filipin (5 *μ*g/mL), EIPA (100 *μ*M), or cytochalasin D (10 *μ*M) for 1 hour before the treatment of the PTD-J-DsRed recombinant protein (40 *μ*g/mL) for 2 hours. The fluorescence values of 100 *μ*L cleared lysate samples were measured (three experiments were performed and the *P* values of filipin, cytochalasin D, and EIPA data relative to the control are 0.0257, 0.000812, and 0.000165, resp.).

**Figure 5:**
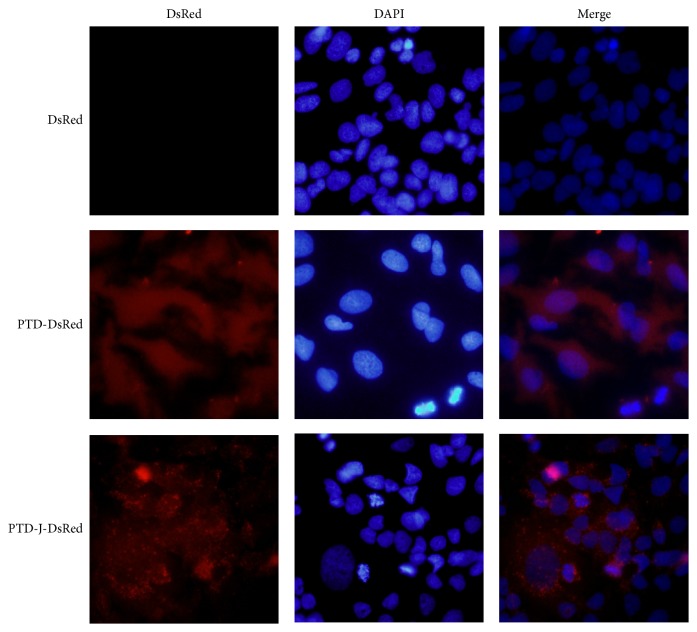
Fluorescence microscopic analysis of the cellular uptake of DsRed, PTD-DsRed, and PTD-J-DsRed recombinant proteins. Huh-7 cells were treated with 40 *μ*g/mL of PTD-DsRed or PTD-J-DsRed recombinant protein for 2 h. After two PBS washes, cells were fixed with 4% paraformaldehyde/PBS for 10 min at room temperature, counterstained with DAPI, and observed under a fluoromicroscope.

It had been suggested that macropinocytosis was involved in the penetration pathway of arginine-rich CPPs [19, 20]. The entrance route of PTD-J-DeRed was analyzed by using inhibitors of different endocytic pathways [26]. The EIPA and cytochalasin D (both are inhibitors on macropinocytosis) and filipin which is an inhibitor of caveolae-mediated endocytosis were tested. As shown in Figure 4, only EIPA and cytochalasin D could reduce the amount of intracellular PTD-J-DsRed indicating that macropinocytosis was involved in its entrance. The same result was obtained for DPV3A-J-DsRed (data not shown). The pattern of PTD-DsRed and PTD-J-DsRed recombinant proteins incorporated into Huh-7 cells was further analyzed using immunofluorescence microscopy. More granular signals were observed in the PTD-J-DsRed image (Figure 5).

## 4. Discussion

### 4.1. PTD-J-DsRed and DPV3A-J-DsRed Recombinant Proteins Were Found in the Medium

During the expression of PTD-J-DsRed and DPV3A-J-DsRed recombinant proteins by* E. coli*, about 10% of total recombinant proteins could be isolated from medium fraction (Figure 2(b)). These “secreted” forms of recombinant proteins were purified by Ni-NTA affinity column and determined with intact N-termini by amino acid sequencing indicating that they are the same as the cellular forms. It is interesting to distinguish how PTD-J-DsRed and DPV3A-J-DsRed recombinant proteins can be released from* E. coli* cells into the medium. Proteins located outside the inner membrane of* E. coli* are usually synthesized with N-terminal signal peptides to target them to either Sec [27, 28] or Tat (twin-arginine translocation) [29, 30] protein export pathway. The major difference between these two export systems is that the Sec apparatus translocates unfolded polypeptides across the membrane, whereas the Tat complex transports already folded proteins. The Tat pathway can transport a heterooligomeric protein complex in which only one subunit possesses a Tat-targeting signal peptide through membrane at once. For example, only the small HybO subunit of HybOC hydrogenase 2 complex has the Tat-targeting signal peptide. The large HybC subunit was transported in complex with the HybO subunit [31]. Another case is the SoxYZ protein complex involved in thiosulfate oxidation. Only SoxY has a Tat-targeting signal peptide and SoxZ is exported in complex with SoxY [29]. Although there is not any datum to support that PTD-J-DsRed or DPV3A-J-DsRed could be carried across cellular membrane by an unknown protein with Tat-targeting signal peptide, it provides a possible pathway to interpret how a protein without signal peptide can be transported across the inner membrane of* E. coli*.

### 4.2. PTD-J-DsRed and DPV3A-J-DsRed Recombinant Proteins Transduce More Effectively Than Their PTD-DsRed and DPV3A-DsRed Counterparts

When Huh-7 cells were treated with the same concentration (40 *μ*g/mL) of recombinant proteins, the amounts of PTD-DsRed and DPV3A-DsRed incorporated were slightly increased with time after 1 hour. However, the maximal amounts of PTD-J-DsRed and DPV3A-J-DsRed within cells were detected around 2 to 4 hours. Then, the fluorescence values decreased because cells began to be lysed (Figure 3(a)). DsRed and J-DsRed could not penetrate into Huh-7 cells. PTD-J-DsRed and DPV3A-J-DsRed could penetrate into Huh-7 cells more effectively than PTD-DsRed and DPV3A-DsRed, respectively, did. These results indicate that the J-domain itself has no cell-penetrating ability; however, it can enhance the cell-penetrating activity of PTD and DPV3A. When Huh-7 cells were treated with 40 *μ*g/mL of PTD-J-DsRed for 2 hours, about 0.6 *μ*g of recombinant proteins was penetrated into 10^4^ cells, corresponding to 10^9^ molecules per cell.

The pattern of PTD-DsRed and PTD-J-DsRed recombinant proteins incorporated into Huh-7 cells was further analyzed using immunofluorescence microscopy. More granular signals were observed in the PTD-J-DsRed image (Figure 5). In addition, the penetration of PTD-J-DsRed recombinant protein into Huh-7 cells was inhibited by EIPA and cytochalasin D (Figure 4). These phenomena indicate that endocytic uptake-endosomal escape pathway may be the major route for PTD-J-DsRed to penetrate into Huh-7 cells. In conclusion, the J-domain can assist CPPs and their cargo to penetrate through the cellular membrane of both* E. coli* and Huh-7 cell more effectively.

## 5. Conclusion

By using red fluorescence protein DsRed as a reporter, the recombinant proteins PTD-J-DsRed and DPV3A-J-DsRed performed higher cell-penetrating activity than PTD-DsRed and DPV3A-DsRed, respectively. Because both DsRed and J-DsRed recombinant proteins could not penetrate into cells, it is suggested that the J-domain could help cell-penetrating peptides PTD and DPV3A as well as their cargo to penetrate through the cellular membrane more effectively.

## Figures and Tables

**Table 1:** Primers for pET22b-CPP vectors construction.

PTD	Peptide	YGRKK RRQRR R
	Forward primer	**TATG GCT **TAT GGT CGT AAG AAA CGT CGT CAG CGT CGT CGT **GTG GGG ATC CCG**
	Reverse primer	**AATT CGG GAT CCC CAC** ACG ACG ACG CTG ACG ACG TTT CTT ACG ACC ATA** AGC CA**

DPV3A	Peptide	AKKRR RESRK KRRRE S
	Forward primer	**TATG **GCT AAA AAA CGC CGT CGT GAA AGC CGT AAA AAA CGT CGT CGT GAA AGC** GGG ATC CCG**
	Reverse primer	**A ATT CGG GAT CCC **GCA TTC ACG ACG ACG TTT TTT ACG GCT TTC ACG ACG GCG TTT TTT AGC** CA**

E162	Peptide	KTVLL RKLLK LLVRK I
	Forward primer	**TATG **AAA ACC GTG CTG CTG CGT AAA CTG CTG AAA CTG CTG GTG CGT AAA ATC** GGG ATC CCG**
	Reverse primer	**A ATT CGG **GAT CCC GAT TTT ACG CAC CAG CAG TTT CAG CAG TTT ACG CAG CAG CAC GGT TTT** CA**

pVEC	Peptide	LLIIL RRRIR KQAHA HSK
	Forward primer	**TATG **CTG CTG ATT ATT CTG CGT CGT CGC ATT CGT AAA CAG GCC CAT GCC CAT TCT AAA** GGG ATC CCG**
	Reverse primer	**A ATT CGG GAT CCC **TTT AGA ATG GGC ATG GGC CTA TTT ACG AAT GCG ACG ACG CAG AAT AAT CAG** CAG CA**

R11	Peptide	RRRRR RRRRR R
	Forward primer	**TATG **CGC CGT CGT CGT CGC CGT CGT CGC CGT CGT CGT** GGG ATC CCG**
	Reverse primer	**A ATT CGG GAT CCC **ACG ACG ACG GCG ACG ACG GCG ACG ACG ACG GCG** CA**

TP13	Peptide	LNSAG YLLGK ALAAL AKKIL
	Forward primer	**TATG **CTG AAC AGC GCG GGT TAT CTG CTG GGT AAA GCC CTG GCC GCC CTG GCG AAA AAG ATT CTG** GGG ATC CCG**
	Reverse primer	**A ATT CGG GAT CCC **CAG AAT CTT TTT CGC CAG GGC GGC CAG GGC TTT ACC CAG CAG ATA ACC CGC GCT GTT CAG** CA**
